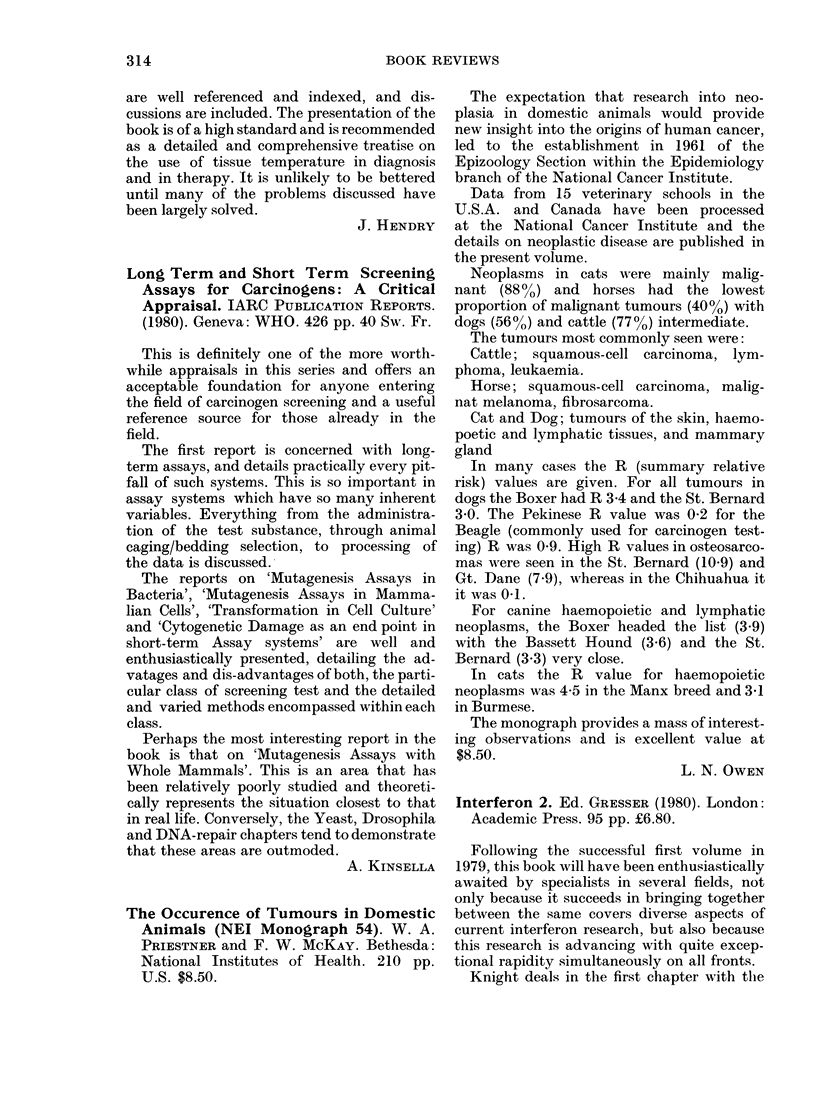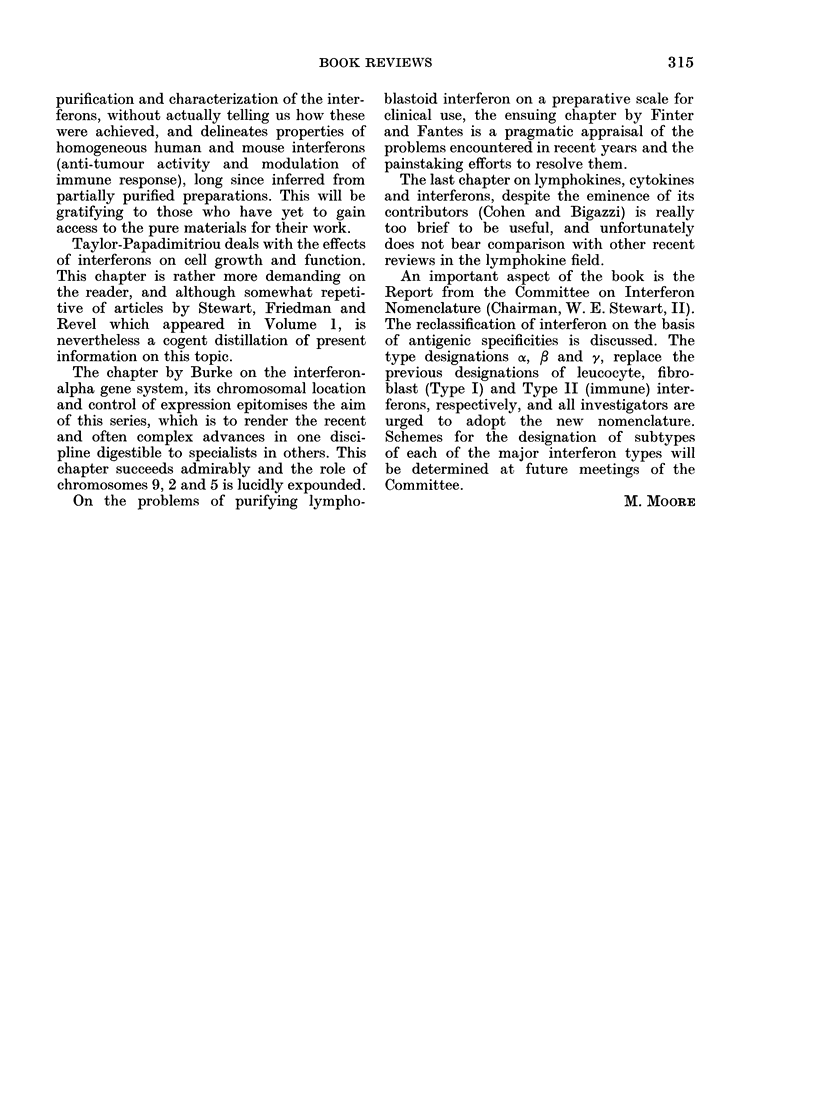# Interferon 2

**Published:** 1981-08

**Authors:** M. Moore


**Interferon 2.** Ed. GRESSER (1980). London: Academic Press. 95 pp. £6.80.

Following the successful first volume in 1979, this book will have been enthusiastically awaited by specialists in several fields, not only because it succeeds in bringing together between the same covers diverse aspects of current interferon research, but also because this research is advancing with quite exceptional rapidity simultaneously on all fronts.

Knight deals in the first chapter with the purification and characterization of the interferons, without actually telling us how these were achieved, and delineates properties of homogeneous human and mouse interferons (anti-tumour activity and modulation of immune response), long since inferred from partially purified preparations. This will be gratifying to those who have yet to gain access to the pure materials for their work.

Taylor-Papadimitriou deals with the effects of interferons on cell growth and function. This chapter is rather more demanding on the reader, and although somewhat repetitive of articles by Stewart, Friedman and Revel which appeared in Volume 1, is nevertheless a cogent distillation of present information on this topic.

The chapter by Burke on the interferon-alpha gene system, its chromosomal location and control of expression epitomises the aim of this series, which is to render the recent and often complex advances in one discipline digestible to specialists in others. This chapter succeeds admirably and the role of chromosomes 9, 2 and 5 is lucidly expounded.

On the problems of purifying lymphoblastoid interferon on a preparative scale for clinical use, the ensuing chapter by Finter and Fantes is a pragmatic appraisal of the problems encountered in recent years and the painstaking efforts to resolve them.

The last chapter on lymphokines, cytokines and interferons, despite the eminence of its contributors (Cohen and Bigazzi) is really too brief to be useful, and unfortunately does not bear comparison with other recent reviews in the lymphokine field.

An important aspect of the book is the Report from the Committee on Interferon Nomenclature (Chairman, W. E. Stewart, II). The reclassification of interferon on the basis of antigenic specificities is discussed. The type designations $\alpha$, $\beta$ and $\gamma$, replace the previous designations of leucocyte, fibroblast (Type I) and Type II (immune) interferons, respectively, and all investigators are urged to adopt the new nomenclature. Schemes for the designation of subtypes of each of the major interferon types will be determined at future meetings of the Committee.

M. Moore